# A detailed error analysis of 13 kernel methods for protein–protein interaction extraction

**DOI:** 10.1186/1471-2105-14-12

**Published:** 2013-01-16

**Authors:** Domonkos Tikk, Illés Solt, Philippe Thomas, Ulf Leser

**Affiliations:** 1 Knowledge Management in Bioinformatics, Computer Science Department, Humboldt-Universität zu Berlin, 10099 Berlin, Germany; 2 Software Engineering Institute, Óbuda University, 1034 Budapest, Hungary; 3 Department of Telecommunications and Telematics, Budapest University of Technology and Economics, 1117 Budapest, Hungary

**Keywords:** Protein–protein interaction, Relation extraction, Kernel methods, Error analysis, Kernel similarity

## Abstract

**Background:**

Kernel-based classification is the current state-of-the-art for extracting pairs of interacting proteins (PPIs) from free text. Various proposals have been put forward, which diverge especially in the specific kernel function, the type of input representation, and the feature sets. These proposals are regularly compared to each other regarding their overall performance on different gold standard corpora, but little is known about their respective performance on the instance level.

**Results:**

We report on a detailed analysis of the shared characteristics and the differences between 13 current methods using five PPI corpora. We identified a large number of rather difficult (misclassified by most methods) and easy (correctly classified by most methods) PPIs. We show that kernels using the same input representation perform similarly on these pairs and that building ensembles using dissimilar kernels leads to significant performance gain. However, our analysis also reveals that characteristics shared between difficult pairs are few, which lowers the hope that new methods, if built along the same line as current ones, will deliver breakthroughs in extraction performance.

**Conclusions:**

Our experiments show that current methods do not seem to do very well in capturing the shared characteristics of positive PPI pairs, which must also be attributed to the heterogeneity of the (still very few) available corpora. Our analysis suggests that performance improvements shall be sought after rather in novel feature sets than in novel kernel functions.

## Background

Automatically extracting protein–protein interactions (PPIs) from free text is one of the major challenges in biomedical text mining [1-6]. Several methods, which usually are co-occurrence-based, pattern-based, or machine-learning based [7], have been developed and compared using a slowly growing body of gold standard corpora [8]. However, progress always has been slow (if measured in terms of precision / recall values achieved on the different corpora) and seems to have slowed down even over the last years; furthermore, current results still do not cope with the performance that has been achieved in other areas of relationship extraction [9].

In this paper, we want to elucidate the reason of the slow progress by performing a detailed, cross-method study of characteristics shared by PPI instances which many methods fail to classify correctly. We concentrate on a fairly recent class of PPI extraction algorithms, namely *kernel methods*[10,11]. The reason for this choice is that these methods were the top-performing in recent competitions [12,13]. In a nutshell, they work as follows. First, they require a training corpus consisting of labeled sentences, some of which contain PPIs and/or non-interacting proteins, while others contain only one or no protein mentions. All sentences in the training corpus are transformed into structured representations that aims to best capture properties of how the interaction is expressed (or not for negative examples). The representations of protein pairs together with their gold standard PPI-labels are analyzed by a kernel-based learner (mostly an SVM), which builds a predictive model. When analyzing a new sentence for PPIs, its candidate protein pairs are turned into the same representation, then classified by the kernel method. For the sake of brevity, we often use the term *kernel* to refer to a combination of SVM learner and a kernel method.

Central to the learning and the classification phases is a so-called kernel function. Simply speaking, a kernel function is a function that takes the representation of two instances (here, protein pairs) and computes their similarity. Kernels functions differ in (1) the underlying sentence representation (bag-of-words, token sequence with shallow linguistic features, syntax tree parse, dependency graphs); (2) the substructures retrieved from the sentence representation to define interactions; and (3) the calculation of the similarity function.

In our recent study [14], we analyzed nine kernel-based methods in a comprehensive benchmark and concluded that dependency graph and shallow linguistic feature representations are superior to syntax tree ones. Although we identified three kernels that outperformed the others (APG, SL, kBSPS; see details below), the study also revealed that none of them seems to be a single best approach due to the sensitivity of the methods to various factors—such as parameter settings, evaluation scenario and corpora. This leads to highly heterogeneous evaluation results indicating that methods are strongly prone to over-fit the training corpus.

The focus of this paper is to perform a cross-kernel error analysis at the instance level with the goal to explore possible ways to improve kernel-based PPI extraction. To this end, we determine difficulty classes of protein pairs and investigate the similarity of kernels in terms of their predictions. We show that kernels using the same input representation perform similarly on these pairs and that building ensembles using dissimilar kernels leads to significant performance gain. Additionally, we identify kernels that perform better on certain difficulty classes; paving the road to more complex ensembles. We also show that with a generic feature set and linear classifiers a performance can be achieved that is on par with most kernels. However, our main conclusion is pessimistic: Our results indicate that significant progress in the field of PPI extraction probably can only be achieved if future methods leave the beaten tracks.

## Methods

We recently performed a comprehensive benchmark of nine kernel-based approaches (hereinafter we refer to them briefly as kernels) [14]. In the meantime, we obtained another four kernels: three of them were originally proposed by Kim *et al.* ([15]) and one is its modification described in [16]; we refer to them collectively as Kim’s kernels. In this work, we investigate similarities and differences between these 13 kernels.

### Kernels

The shallow linguistic (SL) [17] kernel does not use deep parsing information. It is solely based on bag-of-word features (words occurring in the sentence fore-between, between and between-after relative to the pair of investigated proteins), surface features (capitalization, punctuation, numerals), and shallow linguistic (POS-tag, lemma) features generated from tokens left and right to the two proteins (in general: entities) of the protein pair.

Subtree (ST; [18]), subset tree (SST; [19]), partial tree (PT; [20]) and spectrum tree (SpT; [21]) kernels exploits the syntax tree representation of sentences. They differ in the definition of extracted substructures. ST, SST and PT kernels extract subtrees of the syntax parse tree that contain the analyzed protein pair. SpT uses vertex-walks, that is, sequences of edge-connected syntax tree nodes, as the unit of representation. When comparing two protein pairs, the number of identical substructures are calculated as similarity score.

The next group of kernels applies dependency parse sentence representation. Edit distance and cosine similarity kernels (edit, cosine; [22]), as well as the *k*-band shortest path spectrum (kBSPS; [14]) use primarily the shortest path among the entities, but the latter optionally allows for the *k*-band extension of this path in the representation. The most sophisticated kernel, all-path graph (APG; [23]) builds both on the dependency graph and the token sequence representations of the entire sentence, and weighs connections within and outside the shortest path differently.

Kim’s kernels [15] also use the shortest path of the dependency parses. The four kernels differ in the information they use from the parses. The *lexical kernel* uses only lexical information encoded into the dependency tree, that is, nodes are the lemmas of the sentences connected by dependency relation labeled edges. The *shallow kernel* retains only the POS-tag information in the nodes. The similarity score is calculated by both kernels as the number of identical subgraphs of two shortest paths with the specific node labeling. The *combined kernel* is the sum of the former two variants. The *syntactic kernel*, defined in [16], applies exclusively the structural information from the dependency tree, that is, only the edge labels are considered at similarity score calculation.

Since Fayruzov’s implementation of Kim’s kernels does not determine automatically the threshold where to separate positive and negative classes, it has to be specified for each model separately. Therefore, in addition to the parameter search described in [14] and re-used here, we also performed a coarse-grid *threshold searching strategy* in [0,1] with step 0.05. Assuming that the test corpus has similar characteristic as the training one—the usual guess in the absence of further knowledge—we selected the threshold between positive and negative classes such that their ratio approximated the best the ratio measured on the training set. Note that APG [23] applies a similar threshold searching strategy but optimizes the threshold against F-score on the training set.

### Classifiers and parameters

Typically, kernel functions are integrated into SVM implementations. Several freely available and extensible implementations of SVMs exist, among which SVM ^*light*^[24] and LibSVM [25] probably are the most renowned ones. Both can be adapted by supplying a user-defined kernel function. In SVM ^*light*^, kernel functions can be defined as a real function of a pair in the corresponding instance representation. LibSVM, on the other hand, requires the user to pre-compute kernel values, i.e., pass to the SVM learner a matrix containing the pairwise similarity of all instances. Accordingly, most of the kernels we experimented with use the SVM ^*light*^ implementation, except for the SL and Kim’s kernels that use LibSVM, and APG that uses internally a sparse regularized least squares (RLS) SVM.

### Corpora

We use the five freely available and widely used PPI-annotated resources also described in [8], i.e., AIMed [26], BioInfer [27], HPRD50 [28], IEPA [29], and LLL [30].

### Evaluation method

We report on the standard evaluation measures (precision (P), recall (R), F_1_-score (F)). As we have shown in our previous study [14], the AUC measure (area under the receiver operating characteristics curve) that is often used in recent literature to characterize classifiers and independent from the distribution of positive and negative classes, depends very much on the learning algorithm of the classifier, and only partially on the kernel. Therefore, in this study we stick to the above three measures, which actually give a better picture on the expected classification performance on new texts. Results are reported in two different evaluation settings: Primarily, we use the document-level cross-validation scheme (CV), which still seems to be the *de facto* standard in PPI extraction. We also use the cross-learning (CL) evaluation strategy for identifying pairs that behave similarly across various evaluation methods.

In the CV setting, we train and test each kernel on the same corpus using document-level 10-fold cross-validation. We employ the document-level splits used by Airola and many others (e.g., [23,31,32]) to allow for direct comparison of results. The ultimate goal of PPI extraction is the identification of PPIs in biomedical texts with unknown characteristics. This task is better reflected in the CL setting, when training and test sets are drawn from different distributions: in such cases, we train on an ensemble of four corpora and test on the fifth one. CL methodology is generally less biased than CV, where the training and the test data sets have very similar corpus characteristics. Note that the difference in the distribution of positive/negative pairs in the five benchmark corpora (ranging from ∼20 to ∼100%) accounts for a substantial part of the diversity of the performance of approaches [8]. Differences in the annotation of corpora not limited to distribution but also deviates in their annotation guidelines and the definition of what constitutes a PPI; those differences are dominantly kept in the standardized format [8] obtained by applying a transformation approach to yield the greatest common factor in annotations.

### Experimental setup

For the experimental setup we follow the procedure described in [14]. In a nutshell, we applied entity blinding, resolved entity–token mismatch problems and extended the learning format of the sentences with the missing parses. We applied a coarse-grained grid parameter search and selected the best average setting in terms of the averaged F-score measured across the five evaluation corpora as the *default setting* for each kernel.

## Results and discussion

The main goal of our analysis was to better characterize kernel methods and understand their short-comings in terms of PPI extraction. We started by characterizing protein pairs: we divided them into three classes based on their difficulty. Difficulty is defined by the observed classification success level of kernels. We also manually scrutiny some of the pairs that were found to be the most difficult ones, suspecting that the reason for the failure of kernels is in fact an incorrect annotation. We re-labeled a set of such suspicious annotations and re-evaluated if kernels were able to benefit from these modifications. We also compare kernels based on their predictions by defining kernel similarity as prediction agreement on the instance level. We investigate how kernels’ input representations correlate with their similarity. Finally, to quantify the claimed advantage of kernels for PPI extraction, we compare kernels to more simple methods. We used linear, non-kernel based classifiers and a surface feature set also found in the kernel methods.

### Difficulty of individual protein pairs

In this experiment we determine the difficulty of protein pairs. The fewer kernel based approaches are able to classify a pair correctly, the more difficult the pair is. Different kernels’ predictions vary heavily as we have reported in [14]. Here, we show that there exists protein pairs that are inherently difficult to classify (across all 13 kernels), and we investigate whether kernels with generally higher performance classify difficult pairs with greater success.

**Figure 1:**
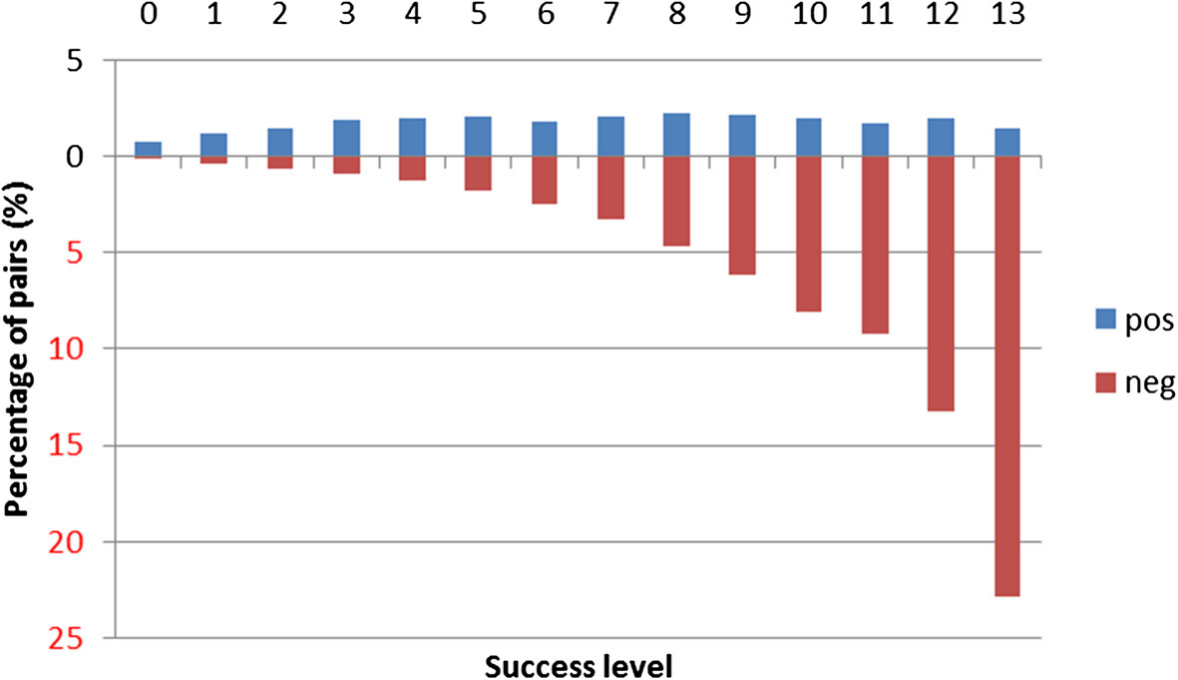
**The distribution of pairs according to classification success level using cross-validation setting.** The distribution of pairs (total, positive and negative) in terms of the number of kernels that classify them correctly (success level) aggregated across the 5 corpora in cross-validation setting. Detailed data for each corpus can be find in Table 1. All 13 kernels are taken into consideration.

**Figure 2:**
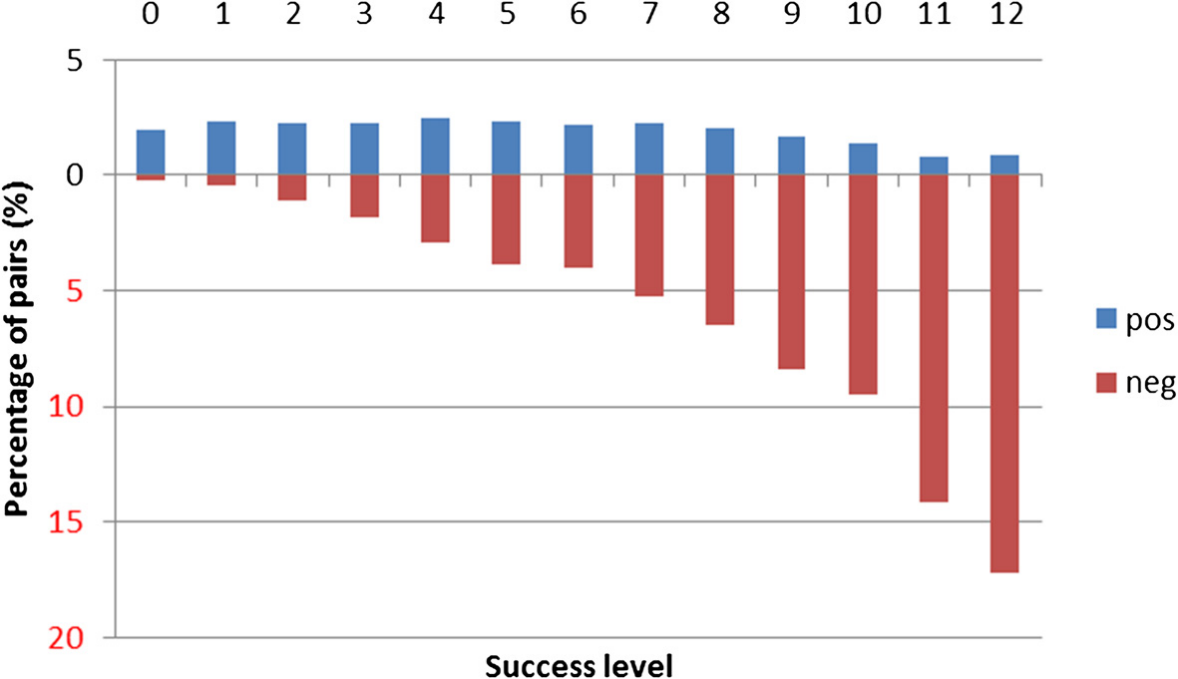
**The distribution of pairs according to classification success level using cross-learning setting.** The distribution of pairs (total, positive and negative) in terms of the number of kernels that classify them correctly (success level) aggregated across the 5 corpora in cross-learning setting. Detailed data for each corpus can be find in Table 2. All kernels except for the very slow PT kernel are taken into consideration.

**Figure 3:**
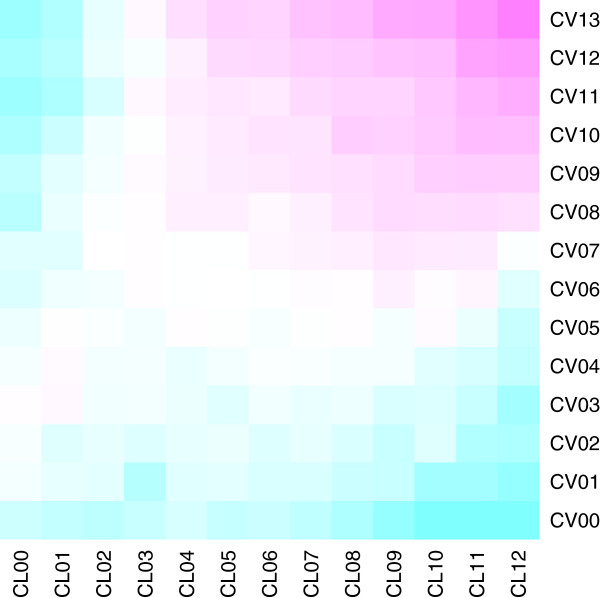
**Heatmap of success level correlation in CV and CL evaluations.** Correlation ranges from 2 (cyan) through 63 (white) to 1266 (magenta) pairs. Hues are on logarithmic scale.

We define the concept of *success level* as the number of kernels being able to classify a given pair correctly. For CV evaluation we performed experiments with all 13 kernels, and therefore have success levels: 0,…,13. For CL evaluation, we omitted the very slow PT kernel (0,…,12). Figures 1 and 2 show the distribution of PPI pairs in terms of success level for CV and CL evaluation aggregated across the 5 corpora, respectively. We also show the same statistics for each corpus separately (Tables 1 and 2). Figure 3 shows the correlation between success levels of CV and CL.

**Table 1:** The distribution of pairs for each corpus according to classification success level using cross-validation setting

	**AIMed**					**BioInfer**					**HPRD50**					**IEPA**					**LLL**				
	**Total**	**T**	**F**	**T, %**	**F, %**	**Total**	**T**	**F**	**T, %**	**F, %**	**Total**	**T**	**F**	**T, %**	**F, %**	**Total**	**T**	**F**	**T, %**	**F, %**	**Total**	**T**	**F**	**T, %**	**F, %**
0	77	73	4	7.3%	0.1%	58	44	14	1.7%	0.2%	4	1	3	0.6%	1.1%	2	1	1	0.3%	0.2%	5	0	5	0.0%	3.0%
1	95	89	6	8.9%	0.1%	158	107	51	4.2%	0.7%	7	4	3	2.5%	1.1%	13	5	8	1.5%	1.7%	7	0	7	0.0%	4.2%
2	105	101	4	10.1%	0.1%	206	130	76	5.1%	1.1%	12	8	4	4.9%	1.5%	11	3	8	0.9%	1.7%	27	0	27	0.0%	16.3%
3	121	104	17	10.4%	0.4%	306	198	108	7.8%	1.5%	18	7	11	4.3%	4.1%	26	13	13	3.9%	2.7%	10	0	10	0.0%	6.0%
4	139	115	24	11.5%	0.5%	349	203	146	8.0%	2.0%	26	10	16	6.1%	5.9%	30	10	20	3.0%	4.1%	16	0	16	0.0%	9.6%
5	140	91	49	9.1%	1.0%	440	225	215	8.9%	3.0%	20	12	8	7.4%	3.0%	43	19	24	5.7%	5.0%	21	2	19	1.2%	11.4%
6	142	70	72	7.0%	1.5%	481	209	272	8.2%	3.8%	33	9	24	5.5%	8.9%	61	22	39	6.6%	8.1%	26	1	25	0.6%	15.1%
7	176	65	111	6.5%	2.3%	619	248	371	9.8%	5.2%	35	15	20	9.2%	7.4%	51	20	31	6.0%	6.4%	29	8	21	4.9%	12.7%
8	248	72	176	7.2%	3.6%	785	256	529	10.1%	7.4%	37	9	28	5.5%	10.4%	79	31	48	9.3%	10.0%	19	6	13	3.7%	7.8%
9	372	69	303	6.9%	6.3%	876	245	631	9.7%	8.8%	46	10	36	6.1%	13.3%	99	32	67	9.6%	13.9%	26	15	11	9.1%	6.6%
10	461	47	414	4.7%	8.6%	1067	204	863	8.1%	12.1%	61	33	28	20.2%	10.4%	101	38	63	11.3%	13.1%	31	19	12	11.6%	7.2%
11	619	29	590	2.9%	12.2%	1061	164	897	6.5%	12.6%	49	19	30	11.7%	11.1%	112	46	66	13.7%	13.7%	32	32	0	19.5%	0.0%
12	1002	43	959	4.3%	19.8%	1390	183	1207	7.2%	16.9%	57	13	44	8.0%	16.3%	106	47	59	14.0%	12.2%	45	45	0	27.4%	0.0%
13	2137	32	2105	3.2%	43.5%	1870	118	1752	4.7%	24.6%	28	13	15	8.0%	5.6%	83	48	35	14.3%	7.3%	36	36	0	22.0%	0.0%

The distribution of pairs (total, positive and negative) in terms of the number of kernels that classify them correctly. Results shown for each corpus separately. Aggregated results are shown in Figure 1. All the 13 kernels are taken into consideration.

**Table 2:** The distribution of pairs for each corpus according to classification success level using cross-learning setting

	**AIMed**					**BioInfer**					**HPRD50**					**IEPA**					**LLL**				
	**Total**	**T**	**F**	**T, %**	**F, %**	**Total**	**T**	**F**	**T, %**	**F, %**	**Total**	**T**	**F**	**T, %**	**F, %**	**Total**	**T**	**F**	**T, %**	**F, %**	**Total**	**T**	**F**	**T, %**	**F, %**
0	41	0	41	0.0%	0.8%	319	319	0	12.6%	0.0%	1	0	1	0.0%	0.4%	9	9	0	2.7%	0.0%	3	3	0	1.8%	0.0%
1	73	6	67	0.6%	1.4%	362	362	0	14.3%	0.0%	4	2	2	1.2%	0.7%	19	17	2	5.1%	0.4%	5	4	1	2.4%	0.6%
2	199	26	173	2.6%	3.6%	322	312	10	12.3%	0.1%	7	3	4	1.8%	1.5%	33	32	1	9.6%	0.2%	10	9	1	5.5%	0.6%
3	315	39	276	3.9%	5.7%	303	280	23	11.0%	0.3%	23	10	13	6.1%	4.8%	38	36	2	10.7%	0.4%	19	19	0	11.6%	0.0%
4	489	71	418	7.1%	8.6%	321	260	61	10.3%	0.9%	27	15	12	9.2%	4.4%	48	45	3	13.4%	0.6%	25	25	0	15.2%	0.0%
5	606	84	522	8.4%	10.8%	355	239	116	9.4%	1.6%	27	15	12	9.2%	4.4%	44	32	12	9.6%	2.5%	25	20	5	12.2%	3.0%
6	547	94	453	9.4%	9.4%	400	208	192	8.2%	2.7%	41	22	19	13.5%	7.0%	51	34	17	10.1%	3.5%	26	18	8	11.0%	4.8%
7	725	136	589	13.6%	12.2%	432	190	242	7.5%	3.4%	43	18	25	11.0%	9.3%	63	32	31	9.6%	6.4%	20	7	13	4.3%	7.8%
8	721	132	589	13.2%	12.2%	586	146	440	5.8%	6.2%	52	17	35	10.4%	13.0%	69	35	34	10.4%	7.1%	34	18	16	11.0%	9.6%
9	767	110	657	11.0%	13.6%	737	95	642	3.7%	9.0%	61	18	43	11.0%	15.9%	107	36	71	10.7%	14.7%	34	19	15	11.6%	9.0%
10	574	118	456	11.8%	9.4%	1060	79	981	3.1%	13.8%	50	14	36	8.6%	13.3%	110	13	97	3.9%	20.1%	56	8	48	4.9%	28.9%
11	414	69	345	6.9%	7.1%	1906	29	1877	1.1%	26.3%	52	16	36	9.8%	13.3%	131	6	125	1.8%	25.9%	50	12	38	7.3%	22.9%
12	363	115	248	11.5%	5.1%	2563	15	2548	0.6%	35.7%	45	13	32	8.0%	11.9%	95	8	87	2.4%	18.0%	23	2	21	1.2%	12.7%

The distribution of pairs (total, positive and negative) in terms of the number of kernels that classify them correctly. Results shown for each corpus separately. Aggregated results are shown in Figure 2. All but the PT kernel are considered. (PT is extremely slow and provide below average results).

The 10–15 percentage point difference in F-score between CV and CL settings reported in [14] can be most evidently seen in the slightly better performance of classifiers on difficult pairs in the CV setting. For example, pairs not classified correctly by any kernels in the CL setting (CL00) are most likely correctly classified by some CV classifiers (CV01–CV05), as shown in Figure 3. Not surprisingly, the pairs correctly classified by most classifiers in either of the CV and CL settings correlate well (see upper right corner in Figure 3). The pairs that are difficult in both evaluation settings (D) are reasonable target for further inspection, as improving kernels to better perform on the them would benefit both scenarios; we attempt to characterize such pairs in subsequent Section.

In order to better identify pairs that are difficult or easy to classify correctly, for each corpus, we took the most difficult and the easiest ∼10% of pairs. For this we cut off the set of pairs at such a success level that the resulting subset of pairs is the closest possible to 10%. Ultimately, we define more universal difficulty classes as the intersection of the respective difficulty classes in CV and CL settings, e.g. D=D_CV_∩D_CL_. When ground truth can be considered to be known, we may further define the intuitive subclasses negative difficult (ND), positive difficult (PD), negative easy (NE) and positive easy (PE), respectively.

We investigated whether and in what extent these classes of pairs overlap depending on the evaluation setting (see Table 3). We used the *χ*^2^-test to check if there was a significantly higher overlap between the two sets compared to as if drawn at random. A p-value lower than 0.001 is considered significant. There are only few cases where correlation is not significant; we discuss these cases separately (1) where the ground truth is known (e.g., PD for HPRD50), and (2) where the ground truth is unknown (e.g., D for LLL).

**Table 3:** The overlap of the pairs that are the most difficult and the easiest to classify correctly by the collection of kernels using cross-validation (CV) and cross-learning (CL) settings

**Difficulty class**			**Corpus**						**Total**
** Difficulty**	**GT**	** Class/setting**	**AIMed**	**BioInfer**	**HPRD50**	**IEPA**	**LLL**	**#**	**%**
difficult	unknown	D _CV_	537	1 077	41	82	39	1776	10.4
		D _CL_	628	1 003	35	99	37	1802	10.6
		D =*D*_CV_∩*D*_CL_	105	530	8	28	0	671	3.9
		p-value	10^−10^	10^−281^	**1**0^−2^	10^−8^	**1.0**		
	positive	PD _CV_	162	281	20	32	17	512	12.2
		PD _CL_	142	319	15	26	16	518	12.3
		PD =*PD*_CV_∩*PD*_CL_	61	111	2	9	7	**190**	4.5
		p-value	10^−60^	10^−95^	**1**0^−1^	10^−7^	10^−6^		
	negative	ND _CV_	463	610	37	50	39	1199	9.3
		ND _CV_	557	644	32	37	28	1298	10.1
		ND =*ND*_CV_∩*ND*_CL_	184	295	12	19	11	**521**	4.0
		p-value	10^−76^	10^−204^	10^−6^	10^−15^	10^−4^		
easy	unknown	E _CV_	2137	1870	85	83	36	4211	24.7
		E _CL_	777	2563	45	95	73	3558	20.8
		E =*E*_CV_∩*E*_CL_	464	1017	23	20	4	1528	8.9
		p-value	10^−45^	10^−184^	10^−7^	10^−3^	**1.0**		
	positive	PE _CV_	104	301	26	48	36	515	12.3
		PE _CL_	115	364	29	27	22	557	13.3
		PE =*PE*_CV_∩*PE*_CL_	49	147	6	10	7	**219**	5.2
		p-value	10^−59^	10^−136^	**1**0^−3^	10^−7^	**1**0^−2^		
	negative	NE _CV_	2105	1752	59	94	23	4033	31.3
		NE _CL_	593	2548	32	87	21	3281	25.5
		NE =*NE*_CV_∩*NE*_CL_	440	1014	21	27	8	**1510**	11.7
		p-value	10^−88^	10^−215^	10^−12^	10^−7^	10^−5^		

We also indicated the size of each set, because they vary depending on the size of success level classes. Abbreviations D, E, PD, ND, PE, and NE refer to the set of difficult (unknown class label), easy (unknown class label), positive difficult, negative difficult, positive easy and negative easy pairs, respectively; GT means ground truth. We highlighted with bold the number pairs in the intersection of CV and CL settings. We show the p-value of Fisher’s independence *χ*^2^-test rounded to the closest factor of 10. Bold typesetting indicates that the size of the overlap is too low.

For case (1), the very few exceptions (PD and PE at HPRD50, and PE at LLL) account only for a mere 1% of PD and 6% of PE pairs. We can also see that the larger a corpus, the better CV and CL evaluations “agree” on the difficulty class of pairs: the strongest correlations can be observed at BioInfer and AIMed.

Considering case (2), for LLL, the intersection of difficult pairs in CV and CL happens to be empty. It was shown in [8,14] that kernels tend to preserve the distribution of positive/negative classes from training to test. LLL has a particularly high ratio of positive examples (50% compared to the average of 25% in the other four corpora). Therefore, kernels predict positive pairs easier for LLL at the CV evaluation, in contrast to CL: in CV evaluation, negative pairs are difficult and in CL evaluation positive ones are difficult. These factors and the relatively small size of the LLL corpus (2% of all five corpora) should explain the empty intersection.

We conclude that our method for identifying the difficult and easy pairs of each class finds meaningful subsets of pairs. We identified 521 ND (negative difficult), 190 PD (positive difficult), 1510 NE (negative easy) and 219 PE (positive easy) pairs.

### How kernels perform on difficult and easy pairs

In Table 4 we show how the different kernels perform on the 521 ND pairs. We publish the same results for the PD, NE, and PE pairs, as well as for all four experiments for CL setting (Tables 5, 6, 7, 8, 9, 10 and 11).

**Table 4:** Classification results on the 521 ND pairs with CV evaluation

**Kernel**	***r***	**TN**	***e***	**TN/*e***	**TN/ND**
edit	18.1	305	427	0.71	0.59
lexical	25.0	203	391	0.52	0.39
SST	26.6	186	382	0.49	0.36
APG	25.3	185	389	0.48	0.36
PT	27.9	185	376	0.49	0.36
syntactic	24.4	180	394	0.46	0.35
cosine	24.9	168	391	0.43	0.32
ST	28.0	160	375	0.43	0.30
shallow	24.6	136	393	0.35	0.26
kBSPS	36.6	122	330	0.37	0.23
combined	24.8	117	392	0.30	0.22
SL	30.4	116	363	0.32	0.22
SpT	46.4	88	279	0.32	0.17

Classification results on the 521 ND pairs with CV evaluation (in decreasing order according to the number of successfully classified pairs). Ratio (*r*) refers to the distribution of positive classes predicted by the kernel measured across the 5 corpora; TN is the number of correctly classified ND pairs; *e* is 521·(1−*r*), the expected number of negative class predictions projected onto the 521 ND pairs.

**Table 5:** Classification results on the 521 ND pairs with CL evaluation

**Kernel**	***r***	**TN**	***e***	**TN/*e***	**TN/#ND**
SST	26.9	288	381	0.76	0.55
edit	22.5	279	404	0.69	0.54
ST	29.2	231	369	0.63	0.44
APG	26.9	207	381	0.54	0.40
SL	29.9	177	365	0.48	0.34
lexical	24.5	170	393	0.43	0.33
cosine	26.6	157	382	0.41	0.30
syntactic	26.9	155	381	0.41	0.30
SpT	42.1	142	302	0.47	0.27
combined	26.8	132	381	0.35	0.25
shallow	28.6	127	372	0.34	0.24
kBSPS	37.1	120	328	0.37	0.23

Classification results on the 521 ND pairs with CL evaluation (in decreasing order according to the number of successfully classified pairs). Ratio (*r*) refers to the distribution of positive classes predicted by the kernel measured across the 5 corpora; TN is the number of correctly classified ND pairs; *e* is 521·(1−*r*), the expected number of negative class predictions projected onto the 521 ND pairs.

**Table 6:** Classification results on the 190 PD pairs with CV evaluation

**Kernel**	***r***	**TP**	***e***	**TP/e**	**TP/#PD**
SpT	46.4	71	88	0.81	0.37
PT	27.9	33	53	0.62	0.17
kBSPS	36.6	22	70	0.31	0.12
ST	28.0	19	53	0.36	0.10
SST	26.6	16	51	0.31	0.08
APG	25.3	15	48	0.31	0.08
SL	30.4	15	58	0.26	0.08
syntactic	24.4	14	46	0.30	0.07
edit	18.1	11	34	0.32	0.06
lexical	25.0	9	47	0.19	0.05
shallow	24.6	7	47	0.15	0.04
cosine	24.9	7	47	0.15	0.04
combined	24.8	4	47	0.09	0.02

Classification results on the 190 PD pairs with CV evaluation (in decreasing order according to the number of successfully classified pairs). Ratio (*r*) refers to the distribution of positive classes predicted by the kernel measured across the 5 corpora; TP is the number of correctly classified PD pairs; *e* is 190·*r*, the expected number of negative class predictions projected onto the 190 PD pairs.

**Table 7:** Classification results on the 190 PD pairs with CL evaluation

**Kernel**	***r***	**TP**	***e***	**TP/e**	**TP/#PD**
SpT	42.1	53	80	0.66	0.28
SST	26.9	39	51	0.76	0.21
ST	29.2	28	55	0.51	0.15
SL	29.9	27	57	0.47	0.14
combined	26.8	16	51	0.31	0.08
shallow	28.6	14	54	0.26	0.07
kBSPS	37.1	14	70	0.20	0.07
APG	26.9	9	51	0.18	0.05
edit	22.5	7	43	0.16	0.04
cosine	26.6	4	51	0.08	0.02
syntactic	26.9	2	51	0.04	0.01
lexical	24.5	1	47	0.02	0.01

Classification results on the 190 PD pairs with CL evaluation (in decreasing order according to the number of successfully classified pairs). Ratio (*r*) refers to the distribution of positive classes predicted by the kernel measured across the 5 corpora; TP is the number of correctly classified PD pairs; *e* is 190·*r*, the expected number of negative class predictions projected onto the 190 PD pairs.

**Table 8:** Classification results on the 1510 NE pairs with CV evaluation

**Kernel**	***r***	**TN**	**FN**	***e***
APG	25.3	1510	0	1129
cosine	24.9	1510	0	1134
edit	18.1	1510	0	1237
combined	24.8	1510	0	1135
shallow	24.6	1510	0	1138
syntactic	24.4	1510	0	1142
kBSPS	36.6	1509	1	957
SL	30.4	1508	2	1051
lexical	25.0	1506	4	1133
PT	27.9	1505	5	1089
ST	28.0	1502	8	1088
SST	26.6	1501	9	1108
SpT	46.4	1484	26	810

Classification results on the 1510 NE pairs with CV evaluation (in decreasing order according to the successfully classified pairs). Ratio (*r*) refers to the distribution of positive classes predicted by the kernel measured across the 5 corpora; TN/FN is the number of correctly/incorrectly classified NE pairs; *e* is 1510·(1−*r*), the expected number of negative class prediction projected onto the 1510 NE pairs.

**Table 9:** Classification results on the 1510 NE pairs with CL evaluation

**Kernel**	***r***	**TN**	**FN**	***e***
shallow	28.6	1510	0	1078
combined	26.8	1505	5	1105
APG	26.9	1504	6	1104
SL	29.9	1504	6	1059
lexical	24.5	1501	9	1140
kBSPS	37.1	1494	16	950
edit	22.5	1491	19	1171
cosine	26.6	1490	20	1109
ST	29.2	1489	21	1069
SST	26.9	1484	26	1104
syntactic	26.9	1483	27	1103
SpT	42.1	1429	81	874

Classification results on the 1510 NE pairs with CL evaluation (in decreasing order according to the successfully classified pairs). Ratio (*r*) refers to the distribution of positive classes predicted by the kernel measured across the 5 corpora; TN/FN is the number of correctly/incorrectly classified NE pairs; *e* is 1510·(1−*r*), the expected number of negative class prediction projected onto the 1510 NE pairs.

**Table 10:** Classification results on the 219 PE pairs with CV evaluation

**Kernel**	***r***	**TP**	**FP**	***e***
combined	24.8	218	1	54
APG	25.3	218	1	55
SpT	46.4	218	1	102
kBSPS	36.6	217	2	80
SL	30.4	216	3	67
shallow	24.6	213	6	54
PT	27.9	210	9	61
syntactic	24.4	208	11	53
cosine	24.9	206	13	55
ST	28.0	205	14	61
lexical	25.0	204	15	55
SST	26.6	201	18	58
edit	18.1	192	27	40

Classification results on the 219 PE pairs with CV evaluation (in decreasing order according to the successfully classified pairs). Ratio (*r*) refers to the distribution of positive classes predicted by the kernel measured across the 5 corpora; TP/FP is the number of correctly/incorrectly classified PE pairs; *e* is 219·*r*, the expected number of positive class prediction projected onto the 219 PE pairs.

**Table 11:** Classification results on the 219 PE pairs with CL evaluation

**Kernel**	***r***	**TP**	**FP**	***e***
kBSPS	37.1	218	1	81
combined	26.8	217	2	59
shallow	28.6	205	14	63
SL	29.9	202	17	65
syntactic	26.9	202	17	59
lexical	24.5	196	23	54
APG	26.9	194	25	59
cosine	26.6	181	38	58
SpT	42.1	177	42	92
edit	22.5	154	65	49
ST	29.2	126	93	64
SST	26.9	123	96	59

Classification results on the 219 PE pairs with CL evaluation (in decreasing order according to the successfully classified pairs). Ratio (*r*) refers to the distribution of positive classes predicted by the kernel measured across the 5 corpora; TP/FP is the number of correctly/incorrectly classified PE pairs; *e* is 219·*r*, the expected number of positive class prediction projected onto the 219 PE pairs.

On difficult pairs (ND&PD), the measured number of true negatives (TN) is smaller than expected based on the class distribution of kernels’ prediction. This phenomenon can be attributed to the difficulty of pairs. The same tendency can be observed for easy pairs (PE&NE) in the opposite direction.

The difference in performance between CV and CL settings reported in [14] cannot be observed on ND pairs: kernels tend to create more general models in the CL setting and identify ND pairs with greater success in average. For PD pairs, kernels produce equally low results in both settings. On the other hand, kernels perform far better for easy pairs (both PE&NE) in CV than in CL setting. This shows that the more general CL models do not work so well on easy pairs than the rather corpus specific CV models; that is, the smaller variability in training examples is also reflected in performance of the learnt model.

As for individual kernels, edit kernel shows the best performance for ND pairs both in terms of TNs and relative to its expected performance. This can be attributed to the low probability of the positive class in edit’s prediction, which is also manifested in the below average performance on positive pairs (PD&PE), and the very good results on NE pairs. SpT, which exhibits by far the highest positive class ratio, performs relatively well on PD pairs both in terms of FNs and the expected relative performance (esp. at CV); this kernel shows analog performance pattern on PD and NE pairs. As for the top performing kernels (APG, SL, kBSPS; [14]) APG performs on all pair subsets equally well (above average or among the best), except at CL on positive pairs; SL is always above the average, except at CV on NDs; however kBSPS works particularly well on easy pairs, and pretty bad on difficult ones (esp. on NDs).

We observed that for difficult (D) pairs, some kernels perform equally better independently of the class label: SST (CL and CV) and ST (CL only). However, this advantage cannot be easily exploited unless difficult pairs are identified in advance. Therefore, next we investigate whether difficulty classes can be predicted by observing only obvious surface features.

### Relation between sentence length, entity distance and pair difficulty

**Figure 4:**
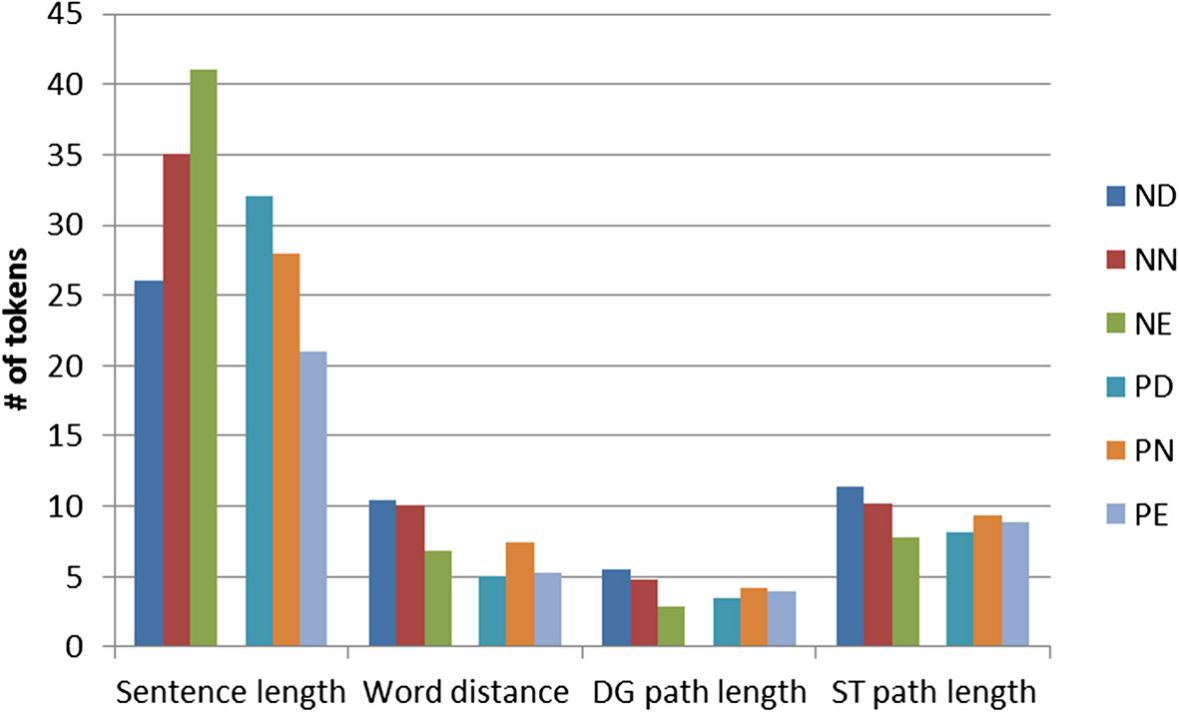
**Characteristics of pairs by difficulty class.** Characteristics of pairs by difficulty class (average sentence length in words, average word distance between entities, average distance in the dependency graph (DG) and syntax tree (ST) shortest path). ND – negative difficult, NN – negative neutral, NE – negative easy, PD – positive difficult, PN – positive neutral, PE – positive easy.

**Figure 5:**
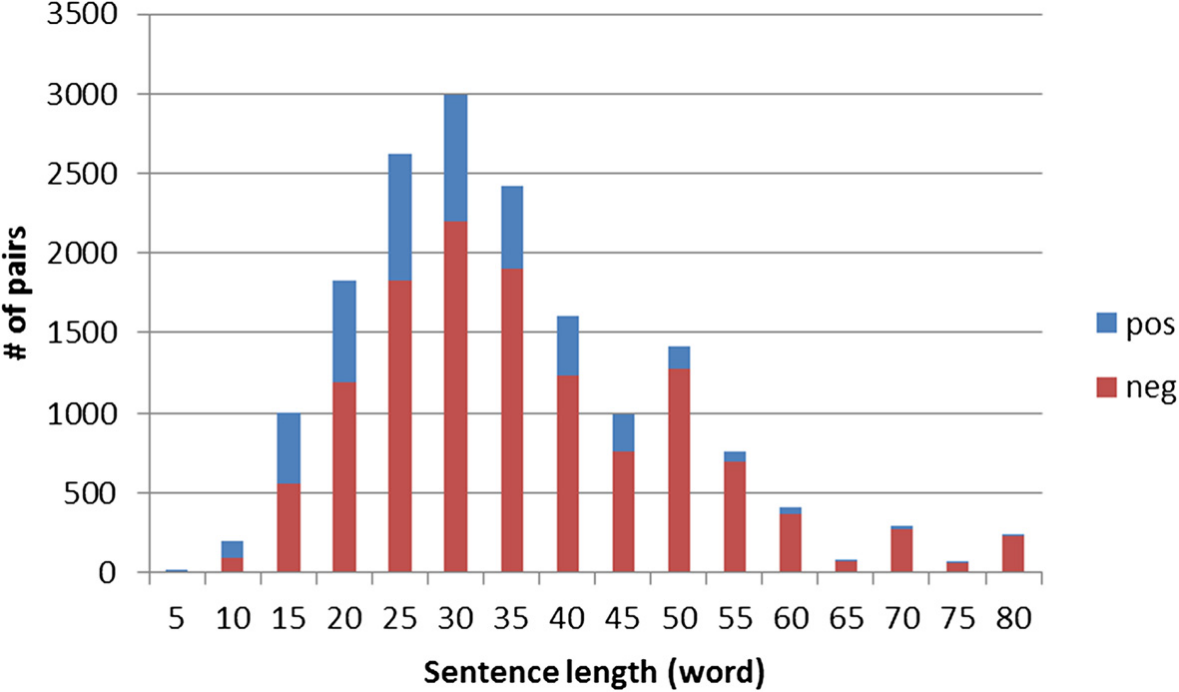
The number of positive and negative pairs vs. the length of the sentence containing the pair.

**Figure 6:**
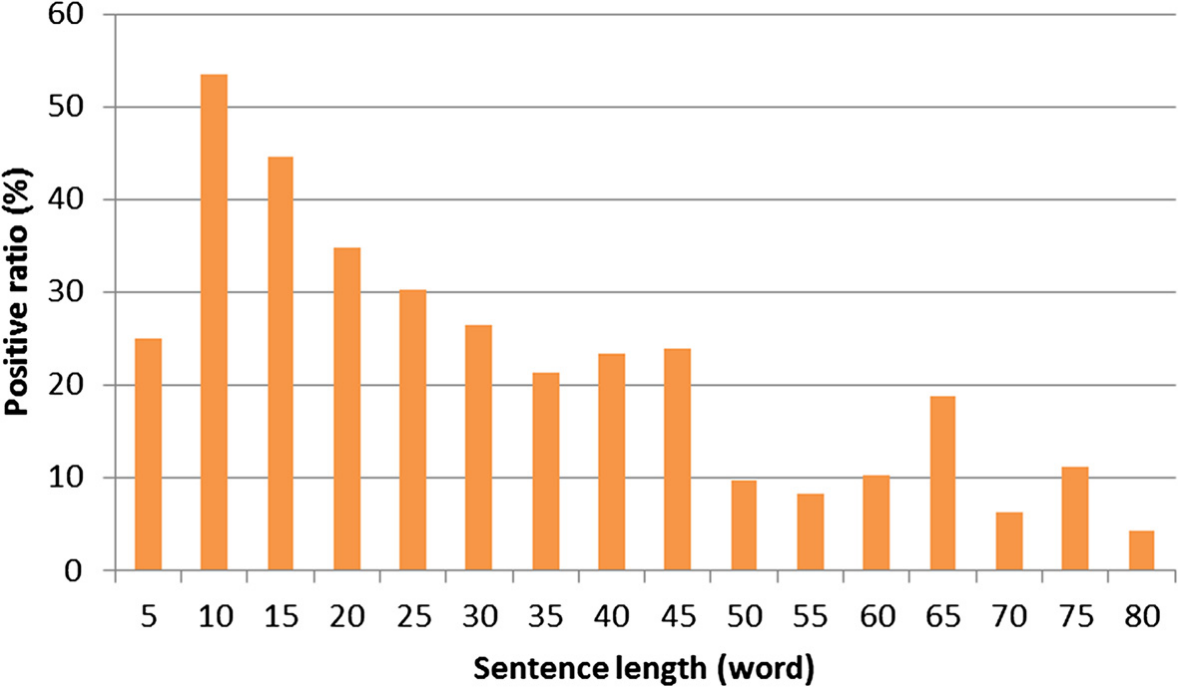
The positive ground truth rate vs. the length of the sentence containing the pair.

In Figure 4 we show the characteristics of sentence difficulty in terms of the average length of the sentence, the average distance between entities, and the size of the shortest path in parse tree. It can be observed that positives pairs are more difficult to classify in longer, and negative pairs in shorter sentences. This correlates with the average length of sentences with positive/negative pairs being 27.6 and 37.2 words – these numbers coincide with the average length of neutral sentences. This is also in accordance with the distribution of positive and negative pairs in terms of the sentence length. Positive pairs occur more often in shorter sentences with less proteins (see Figures 5 and 6), and most of the analyzed classifiers fail to capture the characteristics of rare positive pairs in longer sentences. Long sentences have typically more complicated sentence structure, thus deep parsers are also prone to produce more erroneous parses, which makes the PPI relation extraction task especially difficult.

The distance in words between entities in a sentence seems to be more independent from the difficulty of the pair (see Figure 6). The entities in NE pairs are closer to each other than neutral or more difficult ones, while for positive pairs no such tendency can be observed: the distance in both PE and PD pairs are shorter than at neutral ones. On the other hand, one can observe also at this level that entities of negative pairs are further (9.67) from each others than positives ones (7.15). On the dependency tree level, the difference has a smaller extent: 4.56 (negative) and 4.15 (positive).

We conclude that according to all the three distance measures (word, dependency tree, syntax distance), the farther the entities of negative pairs are located the more difficult are to classify. We also found that positive pairs are typically closer than negative pairs.

Note that similar characteristics were observed at the BioNLP’09 event extraction task regarding the size of minimal subgraph of the dependency graph that includes all triggers and arguments. It was shown in [33] that the size of this subgraph correlates with the class of the event: positive instances are present typically in smaller subgraphs. For the same dataset, in [34] it is shown that the distance between trigger and potential arguments is much smaller for positive than for negative instances.

**Figure 7:**
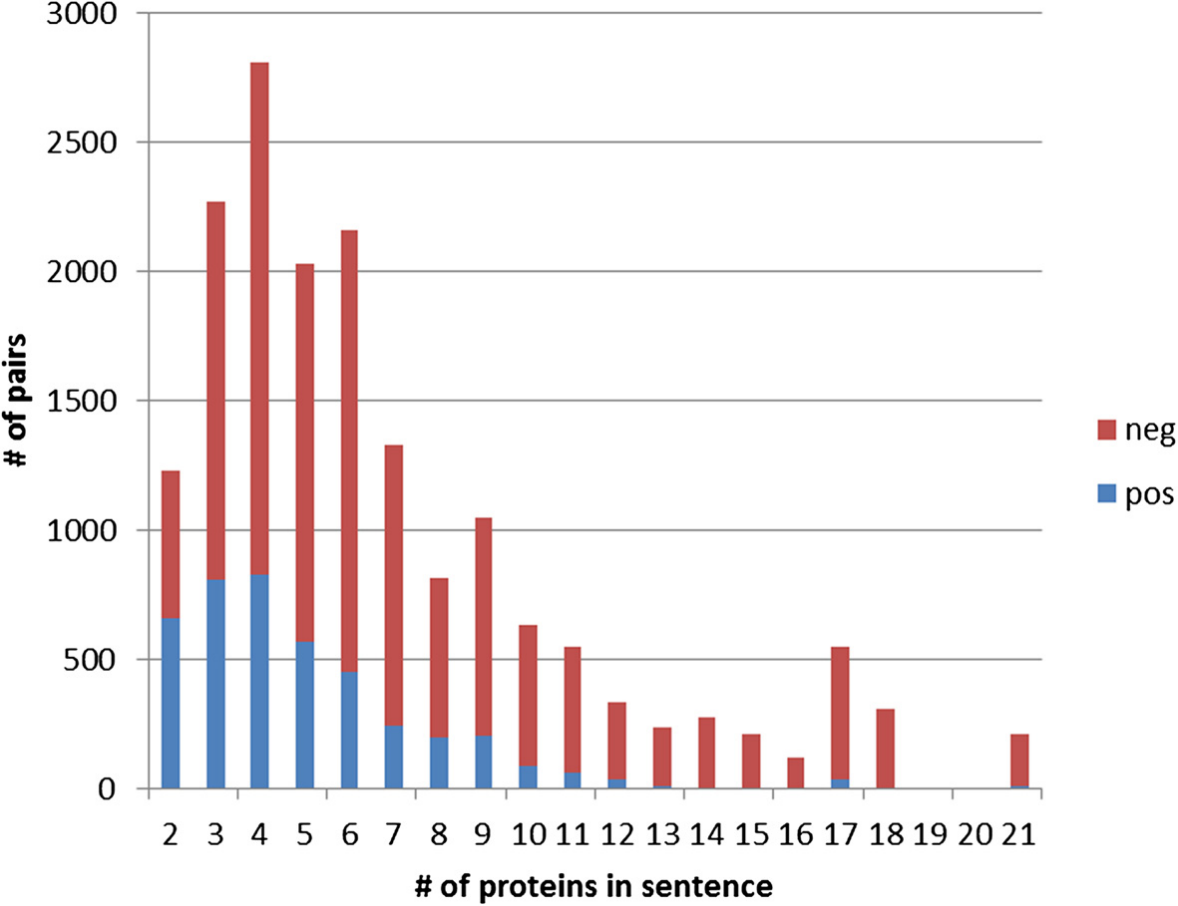
Class distribution of pairs depending on the number of proteins in the sentence.

Next we looked into the relationship between pair difficulty and number of entities in a sentence. In general, long sentences have more protein mentions, and the number of pairs increases quadratically with the number of mentions. We investigated the class distribution of pairs depending on the number of proteins in the sentence (see Figure 7). We can see that the more protein mentions a sentence exhibits, the lower the ratio of positive pairs. This is consistent with the previous experiment on PD pairs: in long sentences there are only few positive pairs, and those are difficult to classify.

Finally, to predict the difficulty class of pairs based on their surface features, we applied a decision tree classifier, results shown in Table 12. We found that predicting the difficult (D) class is particularly hard, with a recall of 20.8 and an F-score of 28.2, indicating that difficult pairs share very few characteristics.

**Table 12:** Classification of difficulty classes based on pair surface features by decision tree

	**Performance**			**Confusion matrix**			
**Difficulty class**	**P**	**R**	**F**_**1**_	**D**	**N**	**E**	**Total**
difficult (D)	43.5	20.8	28.2	148	543	20	711
neutral (N)	92.0	96.2	94.1	178	14 090	372	14 640
easy (E)	72.6	60.0	65.7	14	678	1 037	1 729
Total	88.0	89.4	88.5	*N/A*	*N/A*	*N/A*	

Classification by the Weka J48 classifier. Confusion matrix columns correspond to predicted classes.

Still, we found a number of correlations between pair difficulty and simple surface features that cannot be exploited in kernels as they use a different feature set. Later on, we will show that such features already suffice to build a classifier that is almost *on par* with the state-of-the-art, without using any sophisticated (and costly to compute) kernels.

### Semantic errors in annotation

For some of the very hardest pairs (60 PD and 60 ND), we manually investigated whether their difficulty is actually caused by annotation errors. We identified 23 PD and 28 ND pairs that we considered as incorrectly annotated (for the list of the pair identifiers, see Table 13). Since the selection was drawn from the most difficult pairs, the relatively large number of incorrect annotations does not necessarily make the entire experimentation invalid, though raises the issue of the necessity of a possible re-annotation (see also [35]).

**Table 13:** Incorrectly annotated protein pairs selected from the very hardest positive and negative pairs

**Pair ID**	**GT**	**Type of error**	**Sentence text**
B.d267.s0.p14	T	indirect	However, a number of mammalian DNA repair proteins lack NLS clusters; these proteins include ERCC1, ERCC2 (XPD), mouse RAD51, and the ${\text{HHR23B}}_{{\text{ENT}}_{1}}$/p58 and ${\text{HHR23B}}_{{\text{ENT}}_{2}}$ subunits of XPC.
B.d418.s0.p0	T	functional	Membranous staining and concomitant cytoplasmic localization of E-cadherin, ${\text{alpha-catenin}}_{{\text{ENT}}_{1}}$ and ${\text{gamma-catenin}}_{{\text{ENT}}_{2}}$ were seen in one case with abnormal beta-catenin immunoreactivity.
B.d418.s0.p1	T	functional	Membranous staining and concomitant cytoplasmic localization of ${\text{E-cadherin}}_{{\text{ENT}}_{2}}$, alpha-catenin and ${\text{gamma-catenin}}_{{\text{ENT}}_{1}}$ were seen in one case with abnormal beta-catenin immunoreactivity.
B.d506.s0.p8	T	enumeration	Quantitation of the appearance of X22 banding in primary cultures of myotubes indicates that it precedes that of other myofibrillar proteins and that assembly takes place in the following order: ${\text{X22}}_{{\text{ENT}}_{1}}$, titin, ${\text{myosin heavy chain}}_{{\text{ENT}}_{2}}$, actin, and desmin.
B.d833.s0.p15	T	functional	Within 1 hour of raising the concentration of calcium ions, integrins, cadherins, alpha-catenin, beta-catenin, plakoglobin, vinculin and alpha-actinin appeared to accumulate at cell-cell borders, whereas the focal contact proteins, ${\text{paxillin}}_{{\text{ENT}}_{1}}$ and ${\text{talin}}_{{\text{ENT}}_{2}}$, did not.
B.d833.s0.p14	T	functional	Within 1 hour of raising the concentration of calcium ions, ${\text{integrins}}_{{\text{ENT}}_{2}}$, cadherins, ${\text{alpha-catenin}}_{{\text{ENT}}_{1}}$, beta-catenin, plakoglobin, vinculin and alpha-actinin appeared to accumulate at cell-cell borders, whereas the focal contact proteins, paxillin and talin, did not.
B.d594.s0.p0	T	functional	The clone contains an open reading frame of 139 amino acid residues which shows greater than 40% sequence identity in a 91 amino acid overlap to animal actin-depolymerizing factors (${\text{ADF}}_{{\text{ENT}}_{1}}$), ${\text{cofilin}}_{{\text{ENT}}_{2}}$ and destrin.
B.d296.s2.p20	T	functional	In normal livers, E-cad, ${\text{alpha-catenin}}_{{\text{ENT}}_{2}}$ and beta-catenin, but not CD44s, CD44v5, ${\text{CD44v6}}_{{\text{ENT}}_{1}}$, CD44v7-8, and CD44v10, were expressed at the cell membrane of normal intrahepatic bile ducts.
B.d296.s2.p25	T	functional	In normal livers, E-cad, ${\text{alpha-catenin}}_{{\text{ENT}}_{2}}$ and beta-catenin, but not CD44s, ${\text{CD44v5}}_{{\text{ENT}}_{1}}$, CD44v6, CD44v7-8, and CD44v10, were expressed at the cell membrane of normal intrahepatic bile ducts.
B.d541.s0.p0	T	functional	Since both ${\text{caldesmon}}_{{\text{ENT}}_{2}}$ and ${\text{profilin}}_{{\text{ENT}}_{1}}$ have been found enriched in ruffling membranes of animal cells, our in vitro findings may be relevant to the regulation of actin filaments in living cells.
B.d546.s0.p20	T	functional	Specific antibodies to ${\text{myosin heavy chain}}_{{\text{ENT}}_{2}}$ isoforms (SM1, SM2, ${\text{SMemb}}_{{\text{ENT}}_{1}}$), caldesmon, and alpha-smooth muscle actin and cDNAs for SMemb were used.
A.d28.s234.p1	T	coreference	We have identified a new TNF-related ligand, designated human ${\text{GITR}}_{{\text{ENT}}_{1}}$ ligand (${\text{hGITRL}}_{{\text{ENT}}_{2}}$), and its human receptor (hGITR), an ortholog of the recently discovered murine glucocorticoid-induced TNFR-related (mGITR) protein [4].
B.d765.s0.p14	T	enumeration	To determine the relationship between cell cycle regulation and differentiation, the spatiotemporal expression of cyclin A, cyclin B1, cyclin D1, the ${\text{cyclin-dependent kinase inhibitors}}_{{\text{ENT}}_{1}}$ (CKIs) p27 and ${\text{p57}}_{{\text{ENT}}_{2}}$, and markers of differentiating podocytes in developing human kidneys was investigated by immunohistochemistry.
B.d296.s2.p23	T	functional	In normal livers, ${\text{E-cad}}_{{\text{ENT}}_{1}}$, ${\text{alpha-catenin}}_{{\text{ENT}}_{2}}$ and beta-catenin, but not CD44s, CD44v5, CD44v6, CD44v7-8, and CD44v10, were expressed at the cell membrane of normal intrahepatic bile ducts.
B.d267.s0.p18	T	indirect	However, a number of mammalian DNA repair proteins lack NLS clusters; these proteins include ERCC1, ERCC2 (XPD), mouse RAD51, and the HHR23B/${\text{p58}}_{{\text{ENT}}_{1}}$ and ${\text{HHR23A}}_{{\text{ENT}}_{2}}$ subunits of XPC.
B.d833.s0.p35	T	functional	Within 1 hour of raising the concentration of calcium ions, ${\text{integrins}}_{{\text{ENT}}_{2}}$, ${\text{cadherins}}_{{\text{ENT}}_{1}}$, alpha-catenin, beta-catenin, plakoglobin, vinculin and alpha-actinin appeared to accumulate at cell-cell borders, whereas the focal contact proteins, paxillin and talin, did not.
B.d765.s0.p10	T	enumeration	To determine the relationship between cell cycle regulation and differentiation, the spatiotemporal expression of cyclin A, cyclin B1, cyclin D1, the cyclin-dependent kinase inhibitors (${\text{CKIs}}_{{\text{ENT}}_{1}}$) p27 and ${\text{p57}}_{{\text{ENT}}_{2}}$, and markers of differentiating podocytes in developing human kidneys was investigated by immunohistochemistry.
B.d833.s0.p34	T	functional	Within 1 hour of raising the concentration of calcium ions, ${\text{integrins}}_{{\text{ENT}}_{2}}$, cadherins, alpha-catenin, beta-catenin, plakoglobin, ${\text{vinculin}}_{{\text{ENT}}_{1}}$ and alpha-actinin appeared to accumulate at cell-cell borders, whereas the focal contact proteins, paxillin and talin, did not.
B.d506.s0.p4	T	enumeration	Quantitation of the appearance of X22 banding in primary cultures of myotubes indicates that it precedes that of other myofibrillar proteins and that assembly takes place in the following order: X22, titin, ${\text{myosin heavy chain}}_{{\text{ENT}}_{2}}$, ${\text{actin}}_{{\text{ENT}}_{1}}$, and desmin.
B.d833.s0.p7	T	functional	Within 1 hour of raising the concentration of calcium ions, ${\text{integrins}}_{{\text{ENT}}_{2}}$, cadherins, alpha-catenin, ${\text{beta-catenin}}_{{\text{ENT}}_{1}}$, plakoglobin, vinculin and alpha-actinin appeared to accumulate at cell-cell borders, whereas the focal contact proteins, paxillin and talin, did not.
B.d506.s0.p11	T	enumeration	Quantitation of the appearance of X22 banding in primary cultures of myotubes indicates that it precedes that of other myofibrillar proteins and that assembly takes place in the following order: X22, **titin**${}_{\text{EN}T_{1}}$, ${\text{myosin heavy chain}}_{{\text{ENT}}_{2}}$, actin, and desmin.
B.d833.s0.p29	T	functional	Within 1 hour of raising the concentration of calcium ions, ${\text{integrins}}_{{\text{ENT}}_{2}}$, cadherins, alpha-catenin, beta-catenin, ${\text{plakoglobin}}_{{\text{ENT}}_{1}}$, vinculin and alpha-actinin appeared to accumulate at cell-cell borders, whereas the focal contact proteins, paxillin and talin, did not.
B.d833.s0.p32	T	functional	Within 1 hour of raising the concentration of calcium ions, ${\text{integrins}}_{{\text{ENT}}_{2}}$, cadherins, alpha-catenin, beta-catenin, plakoglobin, vinculin and ${\text{alpha-actinin}}_{{\text{ENT}}_{1}}$ appeared to accumulate at cell-cell borders, whereas the focal contact proteins, paxillin and talin, did not.
A.d60.s528.p0	F	T	The ${\text{v-Raf}}_{{\text{ENT}}_{1}}$ proteins purified from cells infected with EC12 or 22W viruses activated ${\text{MAP kinase}}_{{\text{ENT}}_{2}}$ kinase from skeletal muscle in vitro.
B.d180.s0.p0	F	T	$${\text{DR3}}_{{\text{ENT}}_{2}}$$ signal transduction is mediated by a complex of intracellular signaling molecules including ${\text{TRADD}}_{{\text{ENT}}_{1}}$, TRAF2, FADD, and FLICE.
A.d114.s961.p0	F	T	$${\text{Syntrophin}}_{{\text{ENT}}_{1}}$$ binds to an alternatively spliced exon of ${\text{dystrophin}}_{{\text{ENT}}_{2}}$.
B.d93.s0.p9	F	T	Because ${\text{histone}}_{{\text{ENT}}_{1}}$ H3 shares many structural features with histone H4 and is intimately associated with ${\text{H4}}_{{\text{ENT}}_{2}}$ in the assembled nucleosome, we asked whether H3 has similar functions.
B.d749.s0.p2	F	T	Three actin-associated proteins, actin-binding protein, ${\text{gelsolin}}_{{\text{ENT}}_{1}}$, and profilin, influence gelation, solation, and polymerization, respectively, of ${\text{actin}}_{{\text{ENT}}_{2}}$ in vitro.
B.d639.s0.p0	F	T	The main inhibitory action of p27, a cyclin-dependent kinase inhibitor (${\text{CDKI}}_{{\text{ENT}}_{1}}$), arises from its binding with the cyclin E/${\text{cyclin-dependent kinase 2}}_{{\text{ENT}}_{2}}$ (Cdk2) complex that results in G(1)-S arrest.
B.d334.s0.p0	F	T	In extracts from mouse brain, ${\text{profilin I}}_{{\text{ENT}}_{2}}$ and profilin II can form complexes with regulators of endocytosis, synaptic vesicle recycling and ${\text{actin}}_{{\text{ENT}}_{1}}$ assembly.
A.d141.s1189.p0	F	T	The cyclin-dependent kinase ${\text{Cdk2}}_{{\text{ENT}}_{1}}$ associates with ${\text{cyclins A}}_{{\text{ENT}}_{2}}$, D, and E and has been implicated in the control of the G1 to S phase transition in mammals.
B.d485.s0.p2	F	T	PF4-dependent downregulation of cyclin E-cdk2 activity was associated with increased binding of the ${\text{cyclin-dependent kinase inhibitor}}_{{\text{ENT}}_{1}}$, p21(Cip1/WAF1), to the ${\text{cyclin E}}_{{\text{ENT}}_{2}}$-cdk2 complex.
A.d157.s1329.p4	F	T	Deletion analysis and binding studies demonstrate that a third enzyme, protein kinase C (${\text{PKC}}_{{\text{ENT}}_{1}}$), binds ${\text{AKAP79}}_{{\text{ENT}}_{2}}$ at a site distinct from those bound by PKA or CaN.
A.d60.s529.p0	F	T	Furthermore, a bacterially expressed ${\text{v-Raf}}_{{\text{ENT}}_{1}}$ fusion protein (glutathione S-transferase-p3722W) also activated ${\text{MAP kinase}}_{{\text{ENT}}_{2}}$ kinase in vitro.
A.d199.s1701.p0	F	T	$${\text{Sos}}_{{\text{ENT}}_{1}}$$ in complex with a previously identified 90-kDa protein and designated protein ${\text{80K-H}}_{{\text{ENT}}_{2}}$.
A.d161.s1355.p0	F	T	$${\text{SHPTP2}}_{{\text{ENT}}_{1}}$$ associates with the ${\text{platelet-derived growth factor}}_{{\text{ENT}}_{2}}$ (PDGF) receptor after ligand stimulation, and binding of SHPTP2 to this receptor promotes tyrosine phosphorylation of SHPTP2.
B.d357.s0.p1	F	T	$${\text{Integrin}}_{{\text{ENT}}_{1}}$$ (beta) chains, for example, interact with actin-binding proteins (e.g. ${\text{talin}}_{{\text{ENT}}_{2}}$ and filamin), which form mechanical links to the cytoskeleton.
A.d195.s1663.p2	F	T	Intriguingly, NR1-${\text{calmodulin}}_{{\text{ENT}}_{1}}$ binding is directly antagonized by Ca2+/${\text{alpha-actinin}}_{{\text{ENT}}_{2}}$.
A.d151.s1288.p1	F	T	Immunoprecipitation assays also show a weak substoichiometric association of the ${\text{TATA-binding protein}}_{{\text{ENT}}_{1}}$ (TBP) with ${\text{PTF}}_{{\text{ENT}}_{2}}$, consistent with the previous report of a PTF-related complex (SNAPc) containing substoichiometric levels of TBP and a component (SNAPc43) identical in sequence to the PTF gamma reported here.
B.d485.s0.p4	F	T	PF4-dependent downregulation of cyclin E-cdk2 activity was associated with increased binding of the ${\text{cyclin-dependent kinase inhibitor}}_{{\text{ENT}}_{1}}$, p21(Cip1/WAF1), to the cyclin E-${\text{cdk2}}_{{\text{ENT}}_{2}}$ complex.
B.d814.s0.p26	F	T	We have shown that the ${\text{FH proteins}}_{{\text{ENT}}_{2}}$ Bni1p and Bnr1p are potential targets of the Rho family small GTP-binding proteins and bind to an actin-binding protein, ${\text{profilin}}_{{\text{ENT}}_{1}}$, at their proline-rich FH1 domains to regulate reorganization of the actin cytoskeleton in the yeast Saccharomyces cerevisiae.
B.d14.s0.p4	F	T	Actin-binding proteins such as ${\text{profilin}}_{{\text{ENT}}_{2}}$ and gelsolin bind to phosphatidylinositol (PI) 4,5-bisphosphate (PI 4,5-P2) and regulate the concentration of monomeric ${\text{actin}}_{{\text{ENT}}_{1}}$.
A.d39.s340.p0	F	indirect	Chloramphenicol acetyltransferase assays in F9 cells showed that ${\text{PS1}}_{{\text{ENT}}_{1}}$ suppresses transactivation by ${\text{c-Jun}}_{{\text{ENT}}_{2}}$/c-Jun but not by c-Jun/c-Fos heterodimers, consistent with the reported function of QM/Jif-1.
B.d307.s0.p4	F	indirect	In Acanthamoeba ${\text{actin}}_{{\text{ENT}}_{2}}$ polymerization is regulated, at least in part, by profilin, which binds to ${\text{actin}}_{{\text{ENT}}_{1}}$ monomers, and by capping protein, which both nucleates polymerization and blocks monomer addition at the ’barbed’ end of the filament.
B.d35.s4.p9	F	indirect	We conclude that Aip1p is a ${\text{cofilin}}_{{\text{ENT}}_{2}}$-associated protein that enhances the filament disassembly activity of cofilin and restricts ${\text{cofilin}}_{{\text{ENT}}_{1}}$ localization to cortical actin patches.
L.d35.s1.p1	F	indirect	Our data demonstrate that the ${\text{CtsR}}_{{\text{ENT}}_{1}}$ protein acts as a global repressor of the clpC operon, as well as other class III heat shock genes, by preventing unstressed transcription from either the sigmaB- or ${\text{sigmaA}}_{{\text{ENT}}_{2}}$-dependent promoter and might be inactivated or dissociate under inducing stress conditions.
B.d14.s1.p2	F	indirect	These studies suggest that profilin and ${\text{gelsolin}}_{{\text{ENT}}_{2}}$ may control the generation of 3-OH phosphorylated phosphoinositides, which in turn may regulate the ${\text{actin}}_{{\text{ENT}}_{1}}$ polymerization.
I.d11.s28.p1	F	coreference	The ${\text{phospholipase C}}_{{\text{ENT}}_{1}}$ inhibitor U 71322 prevented the activation of phospholipase C by $${\text{A beta P}}_{{\text{ENT}}_{2}}$$
L.d13.s0.p1	F	indirect	Production of ${\text{sigmaK}}_{{\text{ENT}}_{1}}$ about 1 h earlier than normal does affect Spo0A, which when phosphorylated is an activator of ${\text{sigE}}_{{\text{ENT}}_{2}}$ transcription.
A.d78.s669.p2	F	indirect	Our data suggest that ${\text{TR6}}_{{\text{ENT}}_{1}}$ inhibits the interactions of LIGHT with HVEM / / ${\text{TR2}}_{{\text{ENT}}_{2}}$ and LTbetaR, thereby suppressing LIGHT-mediated HT29 cell death.
B.d223.s0.p9	F	functional	Furthermore, the deletion of ${\text{SJL1}}_{{\text{ENT}}_{2}}$ suppresses the temperature-sensitive growth defect of sac6, a mutant in yeast ${\text{fimbrin}}_{{\text{ENT}}_{1}}$, supporting a role for synaptojanin family members in actin function.

Pair id abbreviations: A – AIMed; B – BioInfer; I – IEPA, L – LLL; ground truth (GT): T (true), F (false); type of errors: indirect – no direct interaction between the entities are described; functional – only functional similarity between entities are described; enumeration – entities are just listed together in an enumeration; coreference – the same protein with different referencing. Entities (in the pair) are highlighted with bold typeface.

We investigated if kernels (we only used APG and SL) could benefit from re-annotation by resetting the ground truth (GT) value of the above 51 pairs and re-running the experiments. Recall that only a mere 0.3% of GT values were changed, most of them in BioInfer (36) and AIMed (12) corpora. We analyzed the performance change both using the original and the re-trained model on the re-annotated corpora (see Table 14). We observed a slight performance improvement using the original model (F-score gain 0.2–0.6). With the re-trained model the performance of APG and SL could be further improved on both corpora (F-score gain 0.25–1.0). This shows that the re-annotation of corpora yield performance uplift even if only a small fraction of pairs is concerned.

**Table 14:** The effect on F-score when changing the ground truth of incorrectly annotated pairs with APG and SL kernels

	**AIMed**					**BioInfer**				
** Kernel**	**Original**	**Modified**	**Retrained**	***Δ***_**m-o**_	***Δ***_**r-m**_	**Original**	**Modified**	**Retrained**	***Δ***_**m-o**_	***Δ***_**r-m**_
APG (setting A)	56.18	56.61	56.14	0.43	−0.47	60.66	60.87	**61.19**	0.21	0.32
APG (setting B)	55.29	55.73	**56.72**	0.44	0.99	60.61	60.83	60.94	0.22	0.11
APG (setting C)	53.20	53.66	53.96	0.46	0.30	59.91	60.36	60.88	0.45	0.52
APG (setting D)	52.30	52.77	52.99	0.47	0.22	59.42	59.90	60.20	0.48	0.30
APG (avg)	54.24	54.69	54.95	0.45	0.26	60.15	60.60	60.80	0.34	0.31
SL	54.48	55.06	**55.57**	0.58	0.51	59.99	60.46	**60.71**	0.47	0.25

Modified – using the original model with modified ground truth; retrained – results of a model retrained on the modified ground truth; *Δ*_m-o_ – difference between modified and original; *Δ*_r-m_ – difference between retrained and modified.

### Similarity of kernel methods

**Figure 8:**
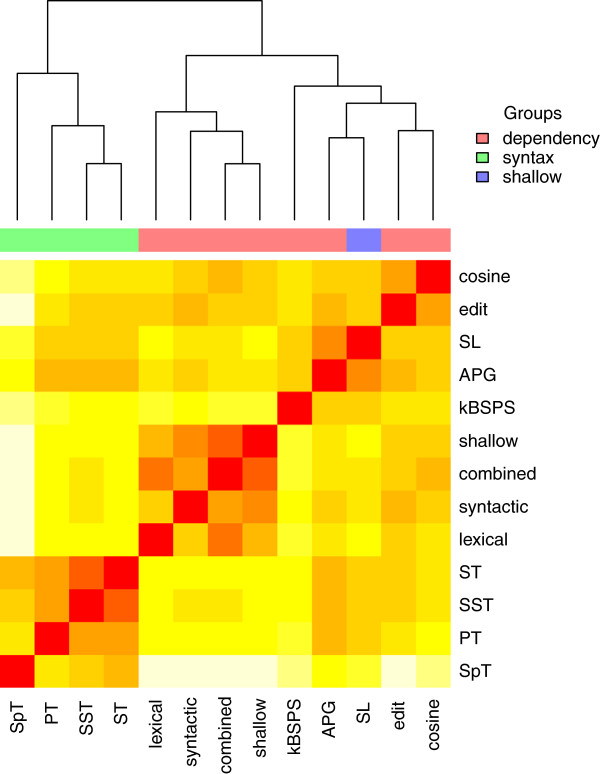
**Similarity of kernels as dendrogram and heat map.** Colors below the dendrogram indicate the parsing information used by a kernel. Similarity of kernel outputs ranges from full agreement (red) to 33% disagreement (yellow) on the five benchmark corpora. Clustering is performed with R’s hclust (http://stat.ethz.ch/R-manual/R-devel/library/stats/html/hclust.html).

Classifier similarity is a key factor when constructing ensemble classifiers. We define the similarity of two kernels as the number of shared annotations versus the total number of annotations. Performing hierarchical clustering with this similarity measure reveals that kernels using the same parsing information group together almost perfectly, i.e., classify pairs much more similarly to each other than to kernels using different parsing information (see Figure 8). Syntax tree based kernels form a clear and separated cluster. Kim’s kernels build a proper sub-cluster within dependency-based kernels. The only kernel that does not use neither dependency nor syntax data, SL, is grouped in the cluster of dependency-based kernels. Interestingly, the outlier in this cluster is kBSPS and not SL. The best two kernels according to [14], APG and SL, are the most similar to each other as they agree on 81% of the benchmark pairs.

Clearly, such characteristics can be exploited in building ensembles as they allow a rationale choice of base classifiers; we will report on using such a strategy in the discussion.

### Feature analysis

To assess the importance of the aforementioned features we constructed a feature space representation of all pairs. We derived surface features from sentences and pairs (see Table 15), including tokens on the dependency graphs (same holds for dependency trees) and syntax tree shortest path, therefore also incorporating parsing information. We then performed feature selection by information gain using each difficulty class as label. The ten most relevant features of the difficult (D) and easy (E) classes are tabulated in Table 16 according to an independent feature analysis. Indicative features of the D-class negatively correlate with the class label: sentence length, the entropy of POS labels along the syntax tree shortest path, number of dependency labels of type *dep* (dependent – fall-back dependency label assigned by the Stanford Parser when no specific label could be retrieved), number of proteins in sentence. The importance of feature *dep* suggests that pairs in sentences having more specific dependency labels are more difficult to correctly predict. For the E class, the entropy of edge labels in the entire syntax tree and dependency graph, and the sentence length correlate positively, while frequency of *nn*, *appos*, *conj_and, dep, det*, etc. correlate negatively.

**Table 15:** Surface and parsing features generated from sentence text used for training non-kernel based classifiers

**Feature type**	**Feature**	**Example**
surface	distance (word/char)	sentence length in characters
		entity distance in words
	count	number of proteins in sentence
	negation clues (s/b/w/a)	negation word before entities
	hedge clues (s/b/w/a)	hedge word after entities
	enumeration clues (b)	comma between entities
	interaction word clues (s/b/w/a)	interaction word in sentence
	entity modifier (a)	-ing word after first entity
parsing	distance (graph)	length of syntax tree shortest path
	occurrence features (entire graph)	number of *conj* constituents in the syntax tree
	occurrence features (shortest path)	number of *conj* constituents along the shortest path in the syntax tree
	frequency features (entire graph)	relative frequency of *conj* labels over the dependency graph
	frequency features (shortest path)	relative frequency of *conj* labels over the shortest path relations
	entropy	Kullback–Leibler divergence of constituent types in the entire syntax tree

Features may refer to both sentence and pair level characteristics. Parsing features were generated from both syntax and dependency parses. Scope of features are typically sentence (s), before entities (b), between entities (w), after entities (a).

**Table 16:** The ten most important features related to difficult (D) and easy (E) classes measured by information gain

	**Difficult (D)**			**Easy (E)**		
**Rank**	** Feature name**	**±**	**IG**	** Feature name**	**±**	**IG**
1	sentence length (char)	−	0.0089	label entropy in ST	+	0.110
2	label entropy in ST (SP)	−	0.0086	sentence length (char)	+	0.090
3	*dep* frequency in DG	−	0.0079	label entropy in DG	+	0.089
4	# of proteins in sentence	−	0.0078	*nn* frequency in DG	−	0.081
5	sentence length (word)	−	0.0069	*appos* frequency in DG	−	0.079
6	*conj_and* frequency in DG	−	0.0069	*conj_and* frequency in DG	−	0.076
7	*prep_with* frequency in DG	−	0.0066	*dep* frequency in DG	−	0.073
8	*prep_with* occurrence in DG	−	0.0066	*det* frequency in DG	−	0.069
9	*nsubjpass* frequency in DG	−	0.0059	*amod* frequency in DG	−	0.063
10	*prep_in* frequency in DG	−	0.0057	*dobj* frequency in DG	−	0.062

IG – information gain; ST – syntax tree; DG – dependency graph; SP – shortest path. Italic typesetting indicates parsing tree labels. The sign after each feature indicates positive/negative correlation.

This experiment justifies that pairs in longer sentences may become more distant and more likely to be negative, thus easier to predict. Several dependency labels are correlated with positive pairs thus their absence render the pair easier to classify (as negative).

### Non-kernel based classifiers

**Figure 9:**
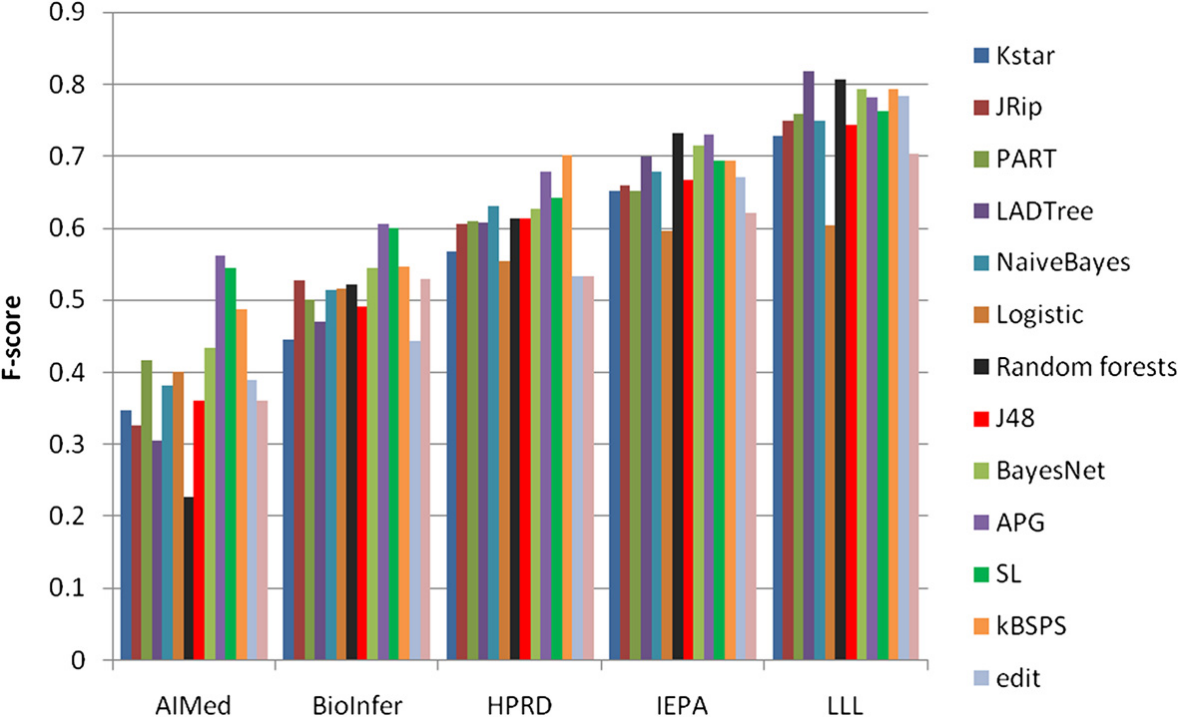
**Comparison of some non-kernel based and kernel based classifiers in terms of F-score (CV evaluation).** The first 9 are non-kernel based classifiers, the last four are kernel based classifiers.

We also compared kernel based classifiers with some linear, non-kernel based classifiers as implemented in Weka [36]. We used the surface feature space created for feature analysis (see Table 15). We ran experiments with 9 different methods (decision trees (J48, LADTree, RandomForest), *k*-NN (KStar), rule learners (JRip, PART), Bayesian (NaiveBayes, BayesNet) and regression methods (Logistic).) We found that the best surface-based classifier, BayesNet, is on par with or better than all kernel based classifiers except APG, SL and kBSPS (see Figure 9). On larger corpora, BayesNet attains 43.4 F-score on AIMed and 54.6 on BioInfer which is outperformed only by the above three kernels. On smaller corpora that are easier to classify having more positive examples, the advantage of kernel based approaches shrinks further.

## Conclusions

In this paper we performed a thorough instance-level comparison of kernel based approaches for binary relation (PPI) extraction on benchmark corpora.

First, we proposed a method for identifying different difficulty classes of protein pairs independently from evaluation setting. Protein interactions are expressed at the linguistic level in diverse ways; its complexity influences the performance of automated methods to classify the pairs correctly. We hypothesized that linguistic complexity of expressing an interaction correlates with classification performance in general, that is, there are PPs on which kernels tend to err independently from the applied evaluation setting (CV or CL). Difficulty classes of PPs were defined based on the success level of kernels in classifying them. We showed that difficulty classes correlate with certain surface features of the pair/the sentence containing the pair, especially word distance, shortest path length between the two proteins in the dependency graph and in the syntax tree. Using these and other surface features, we build linear classifiers that yield results comparable to many of the much more sophisticated kernels. Similar vector space classifiers have been used previously for PPI extraction by [31], however, without an in-depth comparison with existing kernels and in a different evaluation setting. These observations suggest that PPI extraction performance depends far more on the feature set than on the similarity function encoded in kernels, and that future research in the field should focus on finding more expressive features rather than more complex kernel functions. However, it also should be noted that using ever larger feature sets requires considerably more computational resources, considerably increasing the runtime, especially for large-scale experiments. Since the size of currently available training corpora do not keep up with the linguistic diversity, we see two alternatives as possible solutions. The first, computationally more economic strategy focuses on decreasing the linguistic variability using graph rewriting rules on the parse level (see, for instance, [37,38]). Another alternative is to extend available training corpora e.g. by converting certain PPI specific event-level annotations (e.g. regulation, phosphorylation) to PPI annotations in event databases, such as GENIA event data [39]. As an existing example, BioInfer originally also contains richer event information and was transformed to a PPI corpus using some simplifying rules [8].

Second, we built an ensemble by combining three kernels with a simple majority voting scheme. We chose kBSPS, SL and APG as these show above average results across various evaluation settings, but still exhibit considerable disagreement at the instance level (see Figure 8). Combining them leads to a performance improvement of more than 2 percentage points in F-score over the best member’s performance (see Table 17). We also observed a performance increase when combining other kernels, but the results were not on par with that of the better performing kernels, showing that a detailed comparison of kernels in terms of their false positives and false negatives is very helpful for choosing base classifiers for ensembles. Furthermore, we expect that even a higher performance gain can be achieved by employing more sophisticated ensemble construction methods, such as bagging or stacking [40,41]. An alternative approach by [42] was to build a meta-classifier: they classified dependency trees into five different classes depending on the relative position of the verb and the two proteins and learnt a separate classifier for each of these classes.

**Table 17:** Results of some simple majority vote ensembles and comparison with best single methods in terms of F-score

**Combination**	**Corpus**	**P**	**R**	**F**
*Single best*				
APG	AIMed	59.9	53.6	56.2
APG	BioInfer	60.2	61.3	60.7
kBSPS	HPRD50	60.0	**88.4**	70.2
APG	IEPA	66.6	82.6	73.1
kBSPS	LLL	69.9	95.9	79.3
APG+SL+kBSPS	AIMed	58.0	**61.1**	**58.9**
	BioInfer	60.3	66.4	**63.0**
	HPRD50	67.6	76.9	**71.4**
	IEPA	68.6	**85.3**	**75.4**
	LLL	71.7	94.5	80.0
APG+SL+BayesNet	AIMed	55.9	60.3	57.6
	BioInfer	58.6	**68.8**	**63.1**
	HPRD50	**68.4**	69.8	67.7
	IEPA	**71.1**	79.9	74.5
	LLL	**74.3**	92.9	**80.8**
All 13 kernels	AIMed	**67.5**	35.8	46.6
	BioInfer	**61.5**	56.5	58.7
	HPRD50	65.4	69.3	66.1
	IEPA	70.5	78.8	73.7
	LLL	69.6	**98.7**	79.5

Best values are typeset in bold.

Third, the identification of difficult protein pairs was found to be highly useful to spot likely incorrect annotations in the benchmark corpora. We deemed 45% of the 120 manually checked difficult pairs to be incorrectly annotated. We also showed that even very few re-annotated pairs (below 1% of total) influence the kernels’ performance: the re-trained models could generalize the information beyond the affected pairs, and showed a systematic performance gain over the original model. Since our method for finding incorrect annotations is fully automatic, it could be used to decrease the workload of curators at corpus revision.

Overall, we showed that 1–2% of PPI instances are misclassified by all the 13 kernels we considered, independent of which evaluation setting (and hence which training set) was used. Vastly more, 19–30% of PPI instances are misclassified by the majority of these kernels. We also showed that, although a number of features correlate with the “difficulty” of instances, simple combinations of those are not able to tell apart true and false protein pairs. These observations lower the hope that novel types of kernels (using the same input representation) will be able to achieve a breakthrough in PPI extraction performance.

We conclude that one should be rather pessimistic in terms of expecting breakthroughs in kernel-based methods to PPI extraction. Current methods do not seem to do very well in capturing the characteristics shared by positive PPI pairs, which must also be attributed to the heterogeneity of the (still very few) available corpora. We see three main possibilities to escape this situation, some of which have already proven successful in other domains or in other extraction tasks (see references below). For all the three directions we provided below examples found among the 120 examined difficult cases.

The first is to switch focus to more specific forms of interactions, such as regulation, phosphorylation, or complex-building [43,44]. Among the difficult cases it can be observed that incorrectly classified indirect PPIs among the difficult cases (e.g. B.d14.s1.p2, A.d78.s669.p2) tend to be regulatory relationships. As other types of PPIs may be less affected by this issue, the move from generic PPIs to more specific relations should allow for a higher performance for those PPI subtypes. Looking at such more crisply defined problems likely will lead to more homogeneous data and thus raises the chances of classifiers to find the shared characteristics between positive and negative instances, respectively.

Second, we believe that advances could be achieved if methods considered additional background knowledge, for instance by adding them as features of the pair. This encompasses detailed knowledge on the proteins under consideration (like their function, participation in protein families, evolutionary relationships, etc.) and on the semantics of the terms surrounding them. For instance, some false positives pairs were found to contain two proteins that have nearly identical functional properties or that are orthologs. As such co-occurrences are less likely to describe actual interactions, a more informed approach could benefit from taking such aspects into consideration.

Third, pattern-based methods, which are capable of capturing even exotic instances, might be worth looking into again. Even early pattern-based methods are only slightly worse than machine learning approaches [28,45], although those did not fully leverage advances which the NLP community has made especially in terms of telling apart “good” patterns from bad ones [46,47]. Many difficult false positives turned out to be misinterpreted linguistic constructs like enumerations and coreferences. Such constructs might be more appropriately dealt with by using linguistic/syntactical patterns. Note, however, that some other pairs found in sentences with such constructs (e.g. B.d765.s0.p10, A.d28.s234.p1) were correctly annotated by all kernel methods in our assessment. Combining intelligent pattern-selecting with semi-supervised methods for pattern generation [38,48] seems especially promising.

## Abbreviations

PPI: Protein–protein interaction; SVM: Support vector machine; RLS: Regularized least squares; POS-tag: Part-of-speech tag; NLP: Natural language processing; SL: Shallow linguistic kernel; ST: Subtree kernel; SST: Subset tree kernel; PT: Partial tree kernel; SpT: Spectrum tree kernel; edit: Edit distance kernel; cosine: Cosine similarity kernel; kBSPS: k-band shortest path spectrum kernel; APG: All-paths graph kernel; CV: Cross-validation; CL: Cross-learning; T: True; F: False; GT: Ground truth; TP: True positive; TN: True negative; FP: False positive; FN: False negative; D: Difficult; N: Neutral; E: Easy; ND: Negative difficult; PD: Positive difficult; NE: Negative easy; PE: Positive easy; dep: dependent; nn: noun compound modifier; appos: appositional modifier; conj: conjunct.

## Competing interests

The authors declare that they have no competing interests.

## Authors’ contributions

Conceived and designed the experiments: DT, IS, UL. Performed the experiments: DT, IS, PT. Analyzed the data: DT, IS, PT. Wrote the paper: DT, IS, PT, UL. All authors read and approved the final manuscript.
